# Trauma Exposure and Mental Health Problems Among Adults in Central Vietnam: A Randomized Cross-Sectional Survey

**DOI:** 10.3389/fpsyt.2019.00031

**Published:** 2019-02-22

**Authors:** Trang Thi Hanh Do, Ignacio Correa-Velez, Michael P. Dunne

**Affiliations:** ^1^Faculty of Environmental and Occupational Health, Hanoi University of Public Health, Hanoi, Vietnam; ^2^School of Public Health and Social Work, Queensland University of Technology, Brisbane, QLD, Australia; ^3^Institute for Community Health Research, Hue University, Hue, Vietnam

**Keywords:** depression, anxiety, PTSD, mental health, trauma, Vietnam

## Abstract

**Background:** There is relatively little evidence about the psychological and social impacts of trauma exposure in the general population in East Asian countries. Vietnam has a long history of war and poverty, is prone to natural disasters and has high mortality related to traffic accidents. The mental health systems may be inadequate to cope with the resultant trauma.

**Objectives:** This research examines the lifetime prevalence of single and multiple traumas and the association between trauma exposure and depression, anxiety and post-traumatic stress disorder (PTSD) among a randomly selected sample of the adult population in Thua Thien-Hue province in central Vietnam.

**Methods:** Six hundred and eight Vietnamese adults aged 18 years or older participated in the survey. The main tools in the face-to-face interview included the Life Event Checklist (LEC) to measure trauma exposure, the Hospital Anxiety and Depression Scale (HADS) and the PTSD Checklist for DSM-IV (PCL-IV). Hierarchical multiple logistic regression was used to examine associations between trauma exposure and mental health.

**Results:** Forty seven percent of the participants experienced at least one traumatic event in their lifetime and about half of these people were exposed to multiple traumas. The prevalence of depression, anxiety, and PTSD symptoms among the total sample was 12.7, 15.5, and 6.9%, respectively. Prevalence of PTSD among those reporting trauma exposure was 14.8%. Exposure to a higher number of trauma types was associated with increased risk of having depression, anxiety, and PTSD symptoms. Interpersonal traumas were strongly associated with symptoms of all three mental disorders while non-interpersonal traumas were only associated with depressive symptoms.

**Conclusion:** Our findings indicate high burden of lifetime trauma and mental ill health in the adult population of central Vietnam and show a cumulative effect of multiple traumas on symptoms of the three mental disorders. Interpersonal trauma appears to have a more harmful effect on mental health than non-interpersonal trauma. Efforts to improve mental health in Vietnam should focus on reducing risk of preventable interpersonal trauma in every stage of life, and more broadly, ensure greater availability of trauma-sensitive mental health programs and services.

## Introduction

Exposure to traumatic events in a population and the impacts on health and well-being can vary greatly depending on socio-economic, environmental, cultural, and historical factors. The recent World Mental Health Survey revealed that the estimated prevalence of lifetime trauma exposure in the general population of the 24 countries involved in the survey ranges widely, from 29 to 85% (1). Trauma exposure often occurs more frequently in countries with lower income levels (2). This is because of many factors including disparities in living conditions, unstable political situation, ongoing conflicts and lower levels of control over daily life (3). There is convincing evidence, especially from economically developed countries, of a dose-response relationship in which cumulative exposure to traumatic events is associated with progressively increasing risk of psychiatric symptoms including psychological distress, depression, anxiety and Post Traumatic Stress Disorder (PTSD) (4–7).

Over the past few decades, Vietnam has experienced dramatic economic, political and social change through the application of reform policies. This has brought about substantial economic achievements and progress in improving the material well-being of the population (8). However, the reform has also resulted in some negative consequences such as inequity in access to social and health services among groups with different income and geographical backgrounds (9). Moreover, the rapid and uncontrolled industrialization and urbanization has created a trend where people move from rural areas to cities to find better income and educational opportunities. This internal migration has led to weakening of traditional and social family structures and greater inequality in terms of living standards between rural and urban areas (10), and many other negative social phenomena including increased rates of divorce (11), drug and alcohol use (12, 13), and domestic violence (14).

In addition, a wide range of natural disasters from typhoons to floods, landslides, and drought frequently affect Vietnamese people. More than 70% of the national population is vulnerable to risks from these hazards (15). Man-made disasters, especially traffic accidents are also very common in Vietnam. The World Health Organization estimated that in 2013 for every 100,000 Vietnamese people, there were 24.5 road traffic deaths (16). These stressors along with the long history of war and poverty may have contributed to the overall trauma burden and may have substantial impact on the wellbeing in general and mental health in particular of the Vietnamese people.

Current literature documenting the population prevalence of trauma mostly comes from developed countries. The evidence of trauma exposure for less developed countries is still limited (1, 17). To our knowledge, there has not been any study that has explored the prevalence and psychological burden of lifetime trauma exposure among the Vietnamese general population. This paper reports estimates of exposure to a wide range of lifetime traumatic events and experience of multiple traumatic events, and associations with symptoms of depression, anxiety, and PTSD among a randomly selected sample of adults in Thua Thien-Hue province in central Vietnam. In addition to the effect of multiple trauma exposure, this paper examines the effect of different trauma types (interpersonal and non-interpersonal traumas) on the symptoms of mental illness.

## Methods

### Design

We completed a cross-sectional randomized survey of residents in households in rural and urban areas of Thua Thien—Hue province in 2017.

### Participants

Participants were eligible if they were 18 years or older and residing in Thua Thien–Hue province. Six hundred and eight participants took part in the interviews. The sample was selected through several stages. The administrative system in Thua Thien-Hue province has three levels, namely province, district and commune. Thua Thien–Hue province is comprised of nine district-level administrative units, among which three are urban, four are coastal rural and two are mountainous rural districts. At the first stage one urban district, two coastal rural districts and one mountainous rural district were randomly selected from their groups. Second, in each of the selected districts, one commune was randomly selected. Third, in each of the selected communes, 180 households were randomly selected based on the household booklets managed by the commune health center. Finally, in each of these households, a member whose age was 18 years or older was randomly selected. Altogether, there were 720 participants selected and invited to the study with 608 participating in the survey, resulting in a response rate of 84.4%.

### Procedure

Village health collaborators from each of the communes went to the selected participants' houses to give them an invitation letter. Participants were invited to their closest commune health center to be involved in a face-to-face interview with trained research staff, using a structured questionnaire. An interview with each participant was conducted in a private room to ensure privacy and confidentiality. For those who could not go to the commune health center (about 3%), interviews were conducted at their homes. Interviews took an average of 60 minutes. Importantly, the interviewers were not staff of the local commune health center where interviews were done, thus minimizing risks to privacy. Written informed consent was obtained from all participants before the interview. Participation in the study was voluntary. Data were collected from April to June, 2017. Ethics approval was obtained from the Queensland University of Technology Human Research Ethics Committee (Approval No 1600000981) and the Ethical Review Board for Biomedical Research of Hanoi University of Public Health (Approval No 321/2016/YTCC-HD3).

### Instruments

The study used a structured questionnaire which was originally developed in English. The questionnaire was translated into Vietnamese language following a standard back-translation procedure suggested by Brislin (18). In this procedure, forward translation from English to Vietnamese was first performed by a bilingual public health professional. Backward translation (Vietnamese to English) was then undertaken blindly by another bilingual public health professional. A native English speaker later reviewed this version to ensure equivalence to the original questionnaire. For each version of the questionnaire, revision was done when necessary to ensure eventual identical items in the English and Vietnamese versions (18). To further ensure the cultural appropriateness of the questionnaire, consultation with two Vietnamese experts in psychology and psychiatry and with local people was conducted, resulting in rewording of some questions while retaining the content and meaning of the original questionnaire. The key components of the questionnaire included the following:

#### Demographic Characteristics

Variables included age, sex, ethnicity, residence, education, marital status, employment, number of children, and estimated monthly household income. Perceived social status was measured using the MacArthur Scale of Subjective Social Status, which is a 10-rung ladder corresponding numbered from 1 to 10 (19). The top rung represents people who have the highest perceived social status (richest, having highest education level and most respected job) and the bottom rung represents the people who have the lowest social status (poorest, having least education and least respected job or no job) (19).

#### Outcome Variables

##### Symptoms of depression and anxiety

Depression and anxiety were measured with the Hospital Anxiety and Depression Scale (HADS), developed by Zigmond and Snaith (20). The scale is designed to distinguish between depression and anxiety symptoms, comprising seven items for depression and seven items for anxiety. For each item, a 4-point rating scale (0–3), indicating the frequency or severity of the symptoms is used, therefore each subscale can have scores ranging from 0 to 21. Scores from 0 to 7 indicate “no case,” 8–10 “possible case,” and 11–21 “probable case” of depression or anxiety. Although the scale was initially developed for use among patients attending general medical clinics, many studies have confirmed its reliability and validity in community and primary health care populations (21). The scale has been validated and used in surveys in South-east Asia (22, 23). In our study, those who had a total score of 8 or higher for each subscale were classified as having symptoms suggestive of depression/anxiety. This classification of cases was supported by the scale developers (20), numerous other validation studies of the HADS (24) as well as the results of our pilot study in Hue. Data from the present study showed that the two subscales had good internal consistency (Cronbach's alpha = 0.80 for the depression subscale and 0.85 for the anxiety subscale).

##### Post-traumatic stress disorder (PTSD)

PTSD was measured with the Post-traumatic Stress Disorder Checklist (PCL) for DSM-IV (25). This is a 17-item self-report scale designed to assess the full domain of PTSD symptoms according to DSM-IV (including intrusion, numbing/avoidance, and hyper-arousal that correspond to DSM-IV criteria B, C, and D, respectively). In this checklist, respondents are asked how much they were bothered by the symptoms during the past month. Each item has response options ranging from 1 to 5 (not at all to extremely). The PCL is one of the most widely used PTSD self-report instruments (26) and its reliability and validity have been confirmed by numerous studies in different populations around the world (27, 28). In our study, the PCL-S (specific) version was used. It includes questions referring to a specific “stressful experience.” Before being asked questions to assess PTSD symptoms in the PCL-S, the participants are asked about their exposure to traumatic events. PTSD assessment was based on both the total scores of the PCL and DSM-IV symptom criteria. A person was categorized as possibly having PTSD if they had symptomatic response to at least 1 B item (questions 1–5), 3 C items (questions 6–12), and 2 D items (questions 13–17) and a PCL total scores of 30 or higher. The chosen cut point was recommended by the scale's developer—the US National Center for PTSD—for screening PTSD in a general population sample based on a series of validation studies (29). The internal consistency of the PCL in our study was high (Cronbach's alpha = 0.94).

Before the main survey, a pilot study was conducted to validate the outcome measures (the Vietnamese version of the HADS and PCL-IV). The pilot study used the same questionnaire as the main survey and had a sample of 210 adults residing in the same province. Half of these individuals were randomly selected and invited to attend a clinical diagnostic interview to assess depression, anxiety, and PTSD (in a separate interview after having completed the survey questionnaire). The pilot study showed that the HADS and PCL had acceptable reliability and validity and were feasible for screening for depression, anxiety, and PTSD symptoms in Vietnamese contexts.

#### Trauma Exposure

To measure exposure to traumatic events, a list of potential events adopted from the Life Events Checklist (LEC) for DSM-IV was used. The checklist was developed by the US National Center for Post-traumatic Stress Disorder (30). The Checklist includes 16 specific events and an option for other events that are not included in the checklist. For each event, the LEC inquires about multiple types of exposure (1 = happened to me, 2 = witnessed it, 3 = learned about it, 4 = not sure, and 5 = doesn't apply). Psychometric assessment of this checklist in both Western and Asian populations has confirmed that it has adequate reliability and validity (30, 31). In our study, we asked the participants to list only traumatic experiences in which they had witnessed, or were confronted with an event that involved actual or threatened death or serious injury, or a threat to their physical integrity or the physical integrity of others, and that was very frightening and upsetting. The event was considered as endorsed if the participants selected “happened to me” or “witnessed” it or “learned about it.” For each participant, the total number of life-time traumatic events endorsed was calculated and categorized into 4 groups: no exposure, exposure to 1–2 events, exposure to 3–4 events, and exposure to 5 or more events.

#### Covariates

##### Positive mental health

Three positive mental health indicators were assessed: self-esteem, mastery and optimism.

*Self-esteem*. The Rosenberg Self-esteem Scale (RSES) was used to measure self-esteem (32). This ten-item scale asks respondent to rate their agreement with each item (e.g., “I feel I have a number of good qualities” or “At times I think I am no good at all”) on a four-point Likert scale (0 = strongly agree to 3 = strongly disagree). Higher total scores reflect higher level of global self-esteem (32). The scale had been validated and used in several studies in Vietnam (33, 34). In our study, the scale had very good internal consistency (Cronbach's alpha = 0.82).

*Mastery*. Pearlin and Schooler's Self-Mastery Scale (35) was used to measure participants' perception about their ability to deal with life adversities. The scale has 7 items. For each item statement, the respondent is asked to rate their agreement on a 4-point Likert scale (from 1 = strongly disagree to 4 = strongly agree). The total score is obtained by adding up each item score (ranging from 7 to 28) with higher scores reflecting greater level of mastery (35). The scale has been validated and used in Vietnam (34). In our study the scale had good internal consistency (Cronbach's alpha = 0.72).

*Optimism*. Optimism was measured by using the Revised Life Orientation Test. This Test aims to assess generalized expectation for positive as compared to negative life outcomes. The scale includes 10 items, among which four are filler items only and are not to be scored as part of the scale. In this test, the respondent is asked to indicate their level of agreement with items such as “In uncertain times, I usually expect the best” or “I rarely count on good things happening to me” on a 4-point scale ranging from strongly disagree (0) to (1) strongly agree. Reverse coding is required for negatively worded items before adding up all items to obtain the scale's overall score, which ranges from 0 to 24. The higher the overall score, the higher the sense of optimism (36). Studies with both Western and Asian samples have reported favorable validity and reliability of the scale (37, 38). The present study is the first to use it with a Vietnamese sample. Preliminary analyses revealed that our data supported previous studies in conforming with a two-factor structure of the test (37, 38) and we found moderate internal consistency with Cronbach's alpha of 0.62.

*Responsibilities to care for seriously ill or disabled person/people in the family* were assessed by using a single yes/no question, “Do you have to take care of any seriously ill or disabled person in your family?”

##### Number of lifetime chronic diseases

A list of 19 common chronic conditions was included in the questionnaire. The respondents were asked to report all chronic conditions which had been diagnosed by health professionals in their life. The total number of life-time chronic conditions was used for the analyses.

##### Social support

The Multidimensional Scale of Perceived Social Support (MSPSS) was used to measure the level of social support. This scale comprises 12 items referring to social support from different sources: family, friends and significant others. For each item, the respondent was asked to rate on a seven-point scale [from strongly disagree (1) to strongly agree (7)] their level of agreement. Higher total scores represent higher level of perceived social support (39–41). This scale has been validated and used in previous research in Vietnam (34, 42). In our study the scale had excellent internal consistency (Cronbach's alpha = 0.88).

### Statistical Analyses

Descriptive analyses were used to show the distribution of demographic characteristics, trauma exposure and outcome variables. Chi square tests were used to compare differences between males and females in terms of prevalence of the mental disorders and trauma exposure. Hierarchical multiple binary logistic regression was used to examine the relationship between exposure to multiple trauma categories and each of the three mental disorders. A three step process was used for each outcome variable. The first step (Model 1) involved including the demographic characteristics (including gender, age group, ethnicity, residence, marital status, employment, education, household income, number of children, and perceived social status). The second step involved adding multiple trauma categories (Model 2). In the third step, covariates (including responsibilities to care for ill or disabled people in the family, number of life time chronic diseases, self-esteem, optimism, mastery and social support) were all included in the model. Then, manual backward elimination of covariates was performed keeping demographics and multiple traumas in the model (Model 3). Backward elimination was done with those covariates that had a *p* > 0.1. To compare models when excluding covariates, likelihood ratio test was used. Those covariates that changed the coefficients for multiple trauma exposure by more than 15% or resulted in a likelihood ratio test *p* < 0.05 when removed were retained in the final model. As participants not exposed to any traumatic event were not asked for PTSD symptoms, logistic regression analysis for PTSD was restricted to those who were exposed to at least one traumatic event (*N* = 285). All reported *p*-values are two tailed and considered statistically significant at α = 0.05.

The same principles of modeling were applied when the independent effect of trauma types were examined. To examine the effect of trauma types, the 17 lifetime trauma categories in the LEC were grouped into interpersonal traumas and non-interpersonal traumas. Interpersonal traumas encompassed direct exposure (events happening to oneself) to physical assault; assault with a weapon; sexual assault; other unwanted sexual experience; serious injury, harm, or death caused to others; and captivity. Non-interpersonal traumas included witnessing or knowing about physical and sexual assaults and captivity; indirect or direct exposure to disasters, toxic substance, combat or exposure to war zones; life-threatening illness or injury; severe human suffering; sudden violent death; sudden unexpected death of someone close; and the events in the “other” category. This categorization of trauma was informed by previous research involving trauma conceptualization (43, 44). All analyses were performed using STATA version 13.1 (StataCorp, 2013).

## Results

### Participant Characteristics

The mean age of the participants was 42.2 years (*SD* = 15.8 years; range 18–84). As seen in Table 1, twenty three percent were in the older age group (55 years or older), 61.8% were female, and 26.2% belonged to ethnic minority groups. The majority of participants lived in rural areas (74.0%) and were married or living with their partners (80.6%). About one third of the participants were illiterate, and nearly half were living in poor or near-poor households. The participants' perceived social status mean score was 4.1 (*SD* = 1.6; range 1–10). The mean number of children reported by participants was 2.6 (*SD* = 1.9; range 0–10).

**Table 1:** Demographic characteristic of the participants.

**Variable**	***n* (%)**
**Gender (*****N*** **=** **608)**	
Males	232 (38.2)
Females	376 (61.8)
**Age group (*****N*** **=** **598)**	
18-24	75 (12.5)
25-54	385 (64.4)
>55	138 (23.1)
**Ethnicity (*****N*** **= 608)**	
Kinh	449 (73.8)
Minority groups	159 (26.2)
**Residence (*****N*** **= 608)**	
Urban	158 (26.0)
Rural	450 (74.0)
**Marital status (*****N*** **= 608)**	
Never married	65 (10.7)
Married or living as couple	490 (80.6)
Separated/divorced/widowed	53 (8.7)
**Employment (*****N*** **= 608)**	
Unemployed	152 (25.0)
Manual job	348 (57.2)
Non-manual job	108 (17.8)
**Education level (*****N*** **= 608)**	
Illiterate	188 (30.9)
Primary school	176 (29.0)
Secondary School	110 (18.1)
High school or higher	134 (22.0)
**Average household income (*****N*** **= 604)**	
Poor	174 (28.8)
Near-poor	104 (17.2)
None poor	326 (54.0)
	**Mean (*****SD*****)**
**Perceived social status (*****N*** **= 608)**	4.1 (1.6)
**Number of children (*****N*** **= 608)**	2.6 (1.9)

### Prevalence of Mental Disorders

Table 2 presents estimates of the prevalence of depression, anxiety, and PTSD in the study sample. Thirteen percent of the participants reported symptoms suggesting a possible depression diagnosis, and 15.5% reported symptoms suggesting a possible anxiety diagnosis. Of the total population, 6.9% experienced symptoms suggestive of PTSD. The prevalence of PTSD among those who were exposed to one or more traumatic events in their lifetime was 14.8%. Compared to male participants, females had higher prevalence of symptoms of all three mental disorders, though the differences between the two sexes were not statistically significant.

**Table 2:** Prevalence of depression, anxiety, and PTSD symptoms by gender.

	***n*** **(%)**		
	**Male**	**Female**	**Total**
Depression^a^ (*N* = 608)	28 (12.1)	49 (13.0)	77 (12.7)
Anxiety^b^ (*N* = 608)	29 (12.5)	65 (17.3)	94 (15.5)
PTSD^c^ whole sample (*N* = 608)	11 (4.8)	31 (8.3)	42 (6.9)
PTSD^c^ among those reported trauma exposure (*N* = 285)	11 (10.1)	31 (17.8)	42 (14.8)

a *HADS depression subscale score ≥8*.

b *HADS anxiety subscale score ≥8*.

c *PCL score ≥30 and symptomatic response to at least 1 B item (questions 1–5), 3 C items (questions 6–12), and 2 D items (questions 13–17) in the PCL*.

### Exposure to Traumatic Events

Lifetime experience of trauma is detailed in Table 3. Of the total sample, 46.9% reported exposure to some traumatic event in their life. Respondents had experienced an average of 1.1 traumatic events (*SD* = 1.6; range 0–8). Among those who were exposed to at least one trauma type, 59.6% were exposed to multiple trauma types (two or more). Of the total sample, 11.2% reported exposure to two trauma types and 16.8% reported exposure to three or more. There was no significant difference between males and females regarding the total number of traumatic events experienced. The most prevalent trauma experiences were natural disasters (19.9%), transportation accidents (17.8%), and life-threatening illness or injury (15.8%). Males were more likely to report exposure to traumatic events related to fire or explosion, serious accident at work, home, or during recreational activity and combat or exposure to a war-zone. Females were more likely to report events related to unwanted or uncomfortable sexual experience and events categorized in the “other” group (such as canceled wedding and work-related severe stress).

**Table 3:** Prevalence by gender of lifetime trauma.

**Traumatic event**	***n*** **(%)**		
	**Male (*N* = 232)**	**Female (*N* = 376)**	**Total (*N* = 608)**
Natural disaster (for example, flood, typhoon, whirlwind, earthquake)	45 (19.4)	76 (20.2)	121 (19.9)
Fire or explosion^*^	9 (3.9)	5 (1.3)	14 (2.3)
Transportation accident (for example, car accident, boat accident, bus, or motorbike accident)	41 (17.7)	67 (17.8)	108 (17.8)
Serious accident at work, home, or during recreational activity^*^	23 (9.9)	18 (4.8)	41 (6.7)
Exposure to toxic substance (for example, dangerous chemicals, radiation)	7 (3.0)	4 (1.1)	11 (1.8)
Physical assault (for example, being attacked, hit, slapped, kicked, beaten up)	22 (9.5)	26 (6.9)	48 (7.9)
Assault with a weapon (for example, being shot, stabbed, threatened with a knife, gun, bomb)	15 (6.5)	12 (3.2)	27 (4.4)
Sexual assault (rape, attempted rape, made to perform any type of sexual act through force or threat of harm)	1 (0.4)	3 (0.8)	4 (0.7)
Other unwanted or uncomfortable sexual experience^*^	2 (0.9)	15 (4.0)	17 (2.8)
Combat or exposure to a war-zone (in the military or as a civilian)^*^	25 (10.8)	18 (4.8)	43 (7.1)
Captivity (for example, being kidnapped, abducted, held hostage, prisoner of war)	7 (3.0)	5 (1.3)	12 (2.0)
Life-threatening illness or injury	35 (15.1)	61 (16.2)	96 (15.8)
Severe human suffering	14 (6.0)	27 (7.2)	41 (6.7)
Sudden violent death (for example, homicide, suicide)	8 (3.5)	10 (2.7)	18 (3.0)
Sudden unexpected death of someone close	23 (6.1)	20 (8.6)	43 (7.8)
Serious injury, harm, or death you caused to someone else	5 (2.2)	4 (1.1)	9 (1.5)
Any other very stressful event or experience^*^	1 (0.4)	20 (5.3)	21 (3.5)
**Experiencing any trauma**	110 (47.4)	175 (46.5)	285 (46.9)
**NUMBER OF TRAUMA CATEGORIES**			
1	42 (18.1)	73 (19.4)	115 (18.9)
2	28 (12.1)	40 (10.6)	68 (11.2)
3	14 (6.0)	36 (9.6)	50 (8.2)
4	9 (3.9)	10 (2.7)	19 (3.1)
5	6 (2.6)	10 (2.7)	16 (2.6)
6-8	11 (4.7)	6 (1.6)	17 (2.8)

* *p-value comparing males with females < 0.05*.

### Burden of Multiple Trauma Exposure on Symptoms of Depression, Anxiety, and PTSD

Table 4 presents the multiple logistic regression analyses examining the effect of multiple lifetime traumas on depression, anxiety, and PTSD. In the second step of modeling, for all three outcome variables, when multiple trauma exposure was added to model 1 (demographic variables only), it led to a significant improvement of model fit. In Table 4, ORs, 95% CI and *p*-values were reported for those variables retained in the final model. Generally, after controlling for demographic characteristics and covariates, the risk of depression, anxiety, and PTSD was higher as the number of traumatic events increased. Compared to no trauma exposure, the odds of having depression increased by 2.7(*OR* = 2.7; 95%CI 1.3–5.4) for exposure to 1–2 trauma types, by 5.0 (*OR* = 5.0; 95%CI 2.1–11.9) for exposure to 3–4 trauma types, and by 5.9 (*OR* = 5.9; 95%CI 1.9–17.9) for exposure to 5 trauma types or more. A similar trend was seen for anxiety with the odds associated with the trauma exposure categories increasing by 1.8 (*OR* = 1.8; 95%CI 0.9–3.3), 2.8 (*OR* = 2.8; 95%CI 1.2–6.3), and 6.8 (*OR* = 6.8; 95%CI 2.4–19.0), respectively. Compared to those who were exposed to 1–2 trauma types in their life, participants who reported exposure to 3–4 trauma types had a 2.7-fold increased odds (*OR* = 2.7, 95%CI 1.0–7.0) of having PTSD symptoms and those who experienced 5 or more trauma types had a 4.9-fold increased odds (*OR* = 4.9, 95%CI 1.5–15.5) of having PTSD symptoms.

**Table 4:** Summary of multivariable logistic regression for depression, anxiety, and PTSD.

**Source**	**Depression**		**Anxiety**		**PTSD^¶^**	
	**Adjusted OR (95%CI)**	***p*-value**	**Adjusted OR (95%CI)**	***p*-value**	**Adjusted OR (95%CI)**	***p*-value**
**Multiple life time traumas**		0.001^#^		<0.001^#^		0.008^#^
0 type	1		1		–	
1–2 types	2.7 (1.3–5.4)	**0.006**	1.8 (0.9–3.3)	0.081	1	
3–4 types	5.0 (2.1–11.9)	**<0.001**	2.8 (1.2–6.3)	**0.014**	2.7 (1.0–7.0)	**0.042**
5+ types	5.9 (1.9–17.9)	**0.002**	6.8 (2.4–19.0)	**<0.001**	4.9 (1.5–15.5)	**0.008**
**DEMOGRAPHICS**						
**Gender**						
Male	1		1		1	
Female	0.7 (0.3–1.3)	0.198	0.9 (0.5–1.6)	0.642	1.6 (0.6–3.8)	0.330
**Age Group**						
18–24	1		1		1	
25–54	1.0 (0.3–4.0)	0.944	0.6 (0.2–1.7)	0.353	1.1 (0.2–6.0)	0.899
55+	3.0 (0.7–13.5)	0.153	0.2 (0.1–0.9)	**0.029**	0.8 (0.1–5.8)	0.829
**Ethnicity**						
Kinh group	1		1		1	
Minority groups	2.4 (1.1–5.5)	**0.041**	1.4 (0.6–3.0)	0.397	0.8 (0.2–2.4)	0.641
**Residence**						
Urban area	1		1		1	
Rural area	0.8 (0.3–17)	0.519	0.4 (0.2–0.7)	**0.005**	0.6 (0.2–1.7)	0.310
**Marital Status**						
Never married	1		1		1	
Married or living as couple	3.7 (0.4–31.7)	0.238	1.2 (0.4–3.9)	0.807	4.2 (0.4–49.8)	0.253
Separate/divorced/widowed	1.7 (0.2–17.8)	0.637	1.4 (0.3–5.7)	0.668	5.0 (0.4–68.7)	0.228
**Employment**						
Unemployed	1		1		1	
Manual job	0.5 (0.2–1.1)	0.085	0.5 (0.3–1.0)	**0.044**	0.5 (0.2–1.4)	0.195
Non-manual job	1.9 (0.7–5.4)	0.209	1.3 (0.5–3.1)	0.593	1.1 (0.3–4.2)	0.841
**Education Level**						
Illiterate	1		1		1	
Primary school	0.5 (0.2–1.1)	0.094	1.1 (0.6–2.2)	0.770	0.6 (0.2–1.6)	0.288
Secondary School	0.9 (0.4–2.0)	0.761	1.3 (0.6–2.8)	0.532	0.7 (0.2–2.4)	0.580
High school or higher	0.2 (0.0–0.7)	**0.011**	0.6 (0.2–1.6)	0.297	0.4 (0.1–1.7)	0.216
**Household Economic Status**						
Poor	1		1		1	
Near-poor	1.6 (0.7–3.7)	0.244	0.9 (0.4–2.00)	0.791	2.6 (0.8–8.6)	0.122
None poor	0.9 (0.3–2.2)	0.784	0.7 (0.3–1.6)	0.363	1.5 (0.5–5.3)	0.487
**Subjective social status**	0.8 (0.6–1.0)	**0.025**	0.8 (0.6–0.9)	**0.010**	0.8 (0.6–1.2)	0.274
**Number of children**	1.0 (0.8–1.2)	0.831	1.2 (1.0–1.4)	0.093	1.0 (0.8–1.4)	0.843
**COVARIATES**						
**Taking Care Of Sick Or Disabled Person In The Family**						
No	1		1		1	
Yes	2.3 (1.2–4.3)	**0.011**	2.4 (1.4–4.3)	**0.002**	3.7 (1.6–8.7)	**0.002**
**Mastery**	0.7 (0.7–0.9)	**<0.001**	0.8 (0.7–0.9)	**0.003**	–	
**Optimism**	–		–		0.8 (0.7–0.9)	**<0.000**
**Social support**	–		1.0 (0.9–1.0)	**0.006**	–	
**Number of lifetime chronic conditions**	–				–	
0			1			
1–2			1.8 (0.7–4.3)	0.215		
3+			5.0 (1.8–13.7)	**0.002**		

# *p-value for trend, – blank cells: covariates not included in the final model after backward elimination*,

¶ *analysis for PTSD was restricted to those reporting trauma exposure (N = 285). The bold p-values represent significant associations*.

### Effect of Interpersonal and Non-interpersonal Traumas on Symptoms of Depression, Anxiety, and PTSD

Table 5 shows the independent effect of interpersonal traumas and non-interpersonal traumas on symptoms of the three mental illnesses, adjusted for demographics characteristics, the other trauma group, and the covariates retained in the final model. As shown in Table 5, interpersonal traumas were strongly and significantly associated with the symptoms of all three mental illnesses, while non-interpersonal traumas were only associated with an increased risk of depressive symptoms. Compared to those who reported no exposure to interpersonal trauma, those who reported interpersonal trauma had 3.14 times higher odds of having depressive symptoms (*OR* = 3.14, 95%CI: 1.28–7.23), 4.98 times higher odds of having anxiety symptoms (*OR* = 4.98, 95%CI: 2.13–11.64), and 4.43 times higher odds of having PTSD symptoms (*OR* = 4.43, 95%CI: 1.57–12.49). Exposure to non-interpersonal trauma increased the odds of having depressive symptoms by 2.86 times compared to non-exposure (*OR* = 2.86, 95%CI = 1.51–5.44).

**Table 5:** Effect of interpersonal trauma and non-interpersonal trauma on mental health.

**Trauma types**	**Depression^a^**		**Anxiety^b^**		**PTSD^c^**	
	**OR (95%CI)**	***p*-value**	**OR (95%CI)**	***p*-value**	**OR (95%CI)**	***p*-value**
**INTERPERSONAL TRAUMA**						
No	1		1		1	
Yes	3.14 (1.28–7.73)	**0.013**	4.98 (2.13–11.64)	**<0.001**	4.43 (1.58–12.49)	**0.005**
**NON-INTERPERSONAL TRAUMA**						
No	1		1		1	
Yes	2.86 (1.51–5.44)	**0.001**	1.66 (0.93–2.95)	0.088	1.30 (0.15–10.98)	0.809

a *Covariates retained in the final models included responsibilities of caring for a seriously ill or disabled family member and mastery, and perceived social support*.

b *Covariates retained in the final model included responsibilities of caring for a seriously ill or disabled family member, number of chronic diseases, mastery, and perceived social support*.

c *Covariates retained in the final model included responsibilities of caring for a seriously ill or disabled family member and optimism. The bold p-values represent significant associations*.

## Discussion

### Prevalence of Depression, Anxiety, and PTSD

Symptoms of depression, anxiety, and PTSD were found to be common among adults living in central Vietnam. The proportion of adults having symptoms suggestive of depression, anxiety was 12.7 and 15.5%, respectively. These level of psychological morbidity is comparable with normative data for the HADS obtained from studies in other populations [e.g., 8.8–11.0% depression and 15.7–20.1% anxiety in Norway (45), and 16.4% depression in the UK (46). 15.0–19.9% anxiety in the Netherlands (47), and 10.3% depression and 23.4% anxiety in Malaysia (23)]. However, our estimates are much lower when compared to HADS-derived estimates of depression and anxiety among the general population of Korea (43.3% depression and 29.0% anxiety) (48).

PTSD symptoms were identified in 6.9% of the total sample. This prevalence is higher than that reported among non-clinical populations in economically developed countries (4). For example data from the World Mental Health Surveys revealed that the 12-month PTSD symptoms were reported among 3.8% of the total sample in Ireland, 2.5% in the United States and 2.1% in New Zealand (4). The prevalence of PTSD among those who were exposed to at least one traumatic event in our study (14.8%) is within the range of estimates of PTSD among high risk groups including US veterans [between 2 and 17% in a meta-analysis (49)] and among people exposed to natural disasters [between 5.0 and 24.0% in a systematic review (50)].

Many studies find that, compared to men, women tend to report more symptoms of anxiety and depression (51, 52). In contrast, in the present study, although there was a trend for more symptoms of anxiety and PTSD among women than men, these differences were not statistically significant. Similar results in Vietnam were found in a study of post-typhoon prevalence of some psychiatric disorders among adults in Danang and Khanh Hoa provinces (53). This might be explained by culture-specific gender roles in Vietnam, where men are often the household heads and main income earners and may experience more pressure in maintaining employment in an economy that is increasingly competitive (53). It could also be due to the country's long history of war, where men were more likely to be directly exposed to war related traumatic events than were women (53), which is also indicated in the self-reported trauma exposure profile in the current study.

Similar findings of narrow gaps between males and females in some mental illnesses in a number of countries have generated special interest in gender roles (54, 55). It is possible that differences in the prevalence of mental illnesses between males and females arise from difference in the typical stressors faced by each gender, and different coping resources and role-identities between women and men at various points of time throughout history (54, 55). Data from the World Mental Health Survey in Japan revealed higher risk of lifetime mental disorders among men (56). Pooled data from 15 countries participating in the World Mental Health Survey showed that substantial inter-cohort narrowing of the gender gap in major depression was associated with changes in the traditionality of female gender roles (54). Further studies are recommended to examine levels of mental distress and severe disorders between men and women in Vietnam and to identify the factors underlying this pattern.

### Prevalence of Trauma Exposure

To the best of our knowledge, this is the first study to document the co-occurrence of lifetime traumatic events in a general adult population sample in Vietnam. Many participants (nearly 50%) had experienced at least one traumatic event in their lifetime. The World Mental Health Survey showed a wide range in estimates of exposure to traumatic events across the world (30–80%) (1). The prevalence of lifetime trauma exposure in Thua Thien-Hue province is in the same range (about 50–60%) as European countries and Japan (2).

The most prevalent traumatic events were natural disasters (19.9%), transportation accidents (17.8%), and life-threatening illness or injury (15.8%). Participants were asked to report only events that involved “actual or threatened death or serious injury, or a threat to the physical integrity of themselves or others” and which had strong emotional impact on them [as defined in criteria A for exposure to traumatic event for a DSM-IV PTSD diagnosis (57)]. Therefore, the proportion of those who reported natural disaster as a traumatic event may be less than the proportion of those who actually had been exposed to natural disasters but were not personally threatened or harmed. In fact, more than 70% of the Vietnamese population is exposed to natural disaster risks (15). Moreover, road traffic crashes occur very frequently in Vietnam, resulting in a mortality rate of 24.5/100.000 people (16). According to the WHO, injuries related to road traffic are the leading cause of death for those aged 15–29 in the country (58). The frequently reported events related to traffic accident and life-threatening illness or injury in our study reflect the context of the country and highlight their importance in the total burden of trauma among Vietnamese people.

Although our study found that females did not differ significantly from males in the overall risk of trauma exposure, the risk of certain traumas varied between the two sexes. Our results confirm the findings from previous studies that report higher risk for serious accidents and political victimization among males and higher risk for sexual victimization among females (5, 59, 60). These gender differences in trauma exposure suggest that there should be gender-specific interventions and services aimed to lessen the burden of trauma among the Vietnamese population.

### Burden of Trauma Exposure on Mental Health

#### Multiple Trauma Exposure and Symptoms of Depression, Anxiety, and PTSD

Exposure to multiple trauma types has a strong association with symptoms of depression, anxiety, and PTSD in Vietnamese adults. There was a graded association between the number of trauma types and all three measures of mental distress. Those with the highest number of trauma types (5 or more) had a 6-fold increase in the odds of having depressive symptoms and a 7-fold increase in the odds of having anxiety symptoms compared to those with no trauma, and a 5-fold increase in the odds of having PTSD symptoms compared to those exposed to one to two trauma types. Previous research has pointed out that the response of an individual to a more recent trauma may have been exacerbated by an event that happened earlier; in other words, an individual's response may be the result of successive traumatization rather than a given event (61, 62). Our findings lend support to previous studies that demonstrate a strong relationship between incremental levels of trauma and risk of mental ill health (6, 62–64), suggesting that the impact of multiple traumas on mental health is consistent across cultures.

#### Trauma Types and Symptoms of Depression, Anxiety, and PTSD

In addition to the total effect of multiple trauma exposure, the present study provided important evidence regarding the effect of different trauma types on mental health. The strong association between interpersonal trauma and symptoms of the mental illnesses found in this study support prior research that has consistently indicated that, compared to non-interpersonal trauma, exposure to interpersonal trauma bears heavier psychosocial consequences including depression (65–67), anxiety (68), PTSD (43, 66, 69–73), other disorders of extreme stress (DENOS) (74), suicidality (44), and psychiatric externalizing behavior disorder (75). There are several reasons that interpersonal trauma may become remarkably pathogenic. A traumatic event of this type can violate one's assumptions about the world's safety and predictability and can starkly expose the individual to others' capacity to engage in deliberately harmful actions (43). In addition, there has been evidence that traumatic events in which the perpetration involves a person or people with very close relationship, such as abuse by parents or intimate partner violence, are associated with a range of difficulties including affect and behavioral dysregulation and trouble dealing with interpersonal relationships (76). These circumstances may also affect the ability to ensure the ongoing safety of the victims (70).

These findings highlight the importance of the assessment of trauma exposure, particularly those of an interpersonal nature, for the detection and treatment of depression, anxiety, and PTSD among Vietnamese adults. Prevention and treatment of mood disorders may also benefit from assessment of exposure to non-interpersonal trauma. given the increased risk of depressive symptoms found in the present study.

### Positive Beliefs and Symptoms of Depression, Anxiety, and PTSD

This study also provides insight into the association between mental health symptoms and positive beliefs including self-esteem, mastery, and optimism. These three coping resources may enhance the ability to manage stressful events, promote resilience to adversity and lead to better health outcomes (77). Among these coping resources, low mastery and low optimism were found to be significantly associated with symptoms of mental disorder. An increase in one score of the mastery scale was associated with a 30% decrease in the odds of having depressive symptoms, and a 20% decrease in the odds of having anxiety symptoms. An increase in one score of the optimism scale was associated with a 20% decrease in the odds of having PTSD symptoms.

Mastery or sense of control reflects the extent to which an individual feels able to control or influence his or her life events. It also refers to the inner feelings of strength and the ability to overcome difficulties with one's own effort (35, 78, 79). Research into the influence of coping resources has consistently indicated that mastery is strongly linked with better psychological wellbeing (77, 78, 80–85). Optimism facilitates sustained efforts to cope with problems instead of ignoring them or giving up (78). Many studies have found a positive link between optimism and coping with stressful life events and better mental health (78). For example, Applebaum et al. (86) found that there was a negative relationship between the level of optimism and the number of anxiety and depression symptoms and quality of life among patients with advanced cancer.

The findings from this sample of Vietnamese adults support prior studies in confirming that mastery and optimism may predict mental wellbeing and highlight the importance of fostering these positive states of mind in intervention programs that aim to improve mental health.

## Limitations

There were several limitations to this study. First, as the measures of trauma exposure and symptoms of depression, anxiety, and PTSD relied on self-reports, data might have been affected by recall bias. Misclassification might have occurred due to underreporting of traumatic events, mental distress, or both. Past traumas have been found to be minimized in interviews in some previous studies (87). This happens partly because of factors such as embarrassment, shame and social stigma. This key limitation of measurement is shared by most research into trauma that relies on retrospective self-reports that have uncertain validity (88). To reduce the risk to some extent, a standard anonymous questionnaire was used to collect information from all participants and interviews were conducted in private room at commune health centers by research staff who had not had any prior relationship with the participants.

A second limitation is that, due to the small number of people who experienced trauma in some of the 17 specific traumatic event categories, we decided not to examine the independent effect of each event on psychiatric symptoms. Instead, trauma was grouped into two larger groups, namely interpersonal and non-interpersonal trauma. Future studies with larger samples are recommended to examine the effect of cumulative traumas after controlling for the effect of specific trauma types, as well as the independent effect of each trauma type. Third, this is a cross-sectional study therefore, causality inferences could not be established. Finally, as our study was conducted in central Vietnam, a region very prone to natural disasters, annually subjected to heavy loss of human life and property due to floods and typhoons (15), the survey results might not be generalized to other parts of Vietnam. However, the study contributes to current knowledge on trauma exposure and its association with mental disorders in Vietnam, and may be of relevance to other developing countries.

## Conclusions

This is the first study to estimate exposure to multiple traumas in a random community-based sample of adults in Vietnam. Although limited to one province, the findings indicate the strong impact that traumatic events can have on psychological well-being, and suggest the need for more extensive national research. The evidence at hand supports calls for integration of traumatology into mental health care, to thereby broaden the focus of current services that primarily are concerned with diagnosis and management of endogenous mental illnesses.

## Data Availability Statement

The raw data supporting the conclusions of this manuscript will be made available by the authors, without undue reservation, to any qualified researcher.

## Author Contributions

TD, MD, and IC-V developed the study concept. TD collected and analyzed data. All authors were involved in manuscript preparation and approved the final version of this manuscript.

### Conflict of Interest Statement

The authors declare that the research was conducted in the absence of any commercial or financial relationships that could be construed as a potential conflict of interest.
